# Supplementary material for: Non-Clinical Factors Determining the Prescription of Antibiotics by Veterinarians: A Systematic Review
Source: Antibiotics (Basel). 2021 Jan 30;10(2):133. doi: 10.3390/antibiotics10020133 (PMC7912449; doi:10.3390/antibiotics10020133)
Supplement: Supplementary file 1 [file antibiotics-10-00133-s001.pdf]

**Table S1 Study of assessment of quality and susceptibility to bias of quantitative and mixed studies: AXIS tool.**

| Author | Year | AXIS criterion | | | | | | | | | | | | | | | | | | | |
|---|---|---|---|---|---|---|---|---|---|---|---|---|---|---|---|---|---|---|---|---|---|
| | | 1 | 2 | 3 | 4 | 5 | 6 | 7 | 8 | 9 | 10 | 11 | 12 | 13 | 14 | 15 | 16 | 17 | 18 | 19 | 20 |
| Eriksen 55 | 2019 | | | | | | | | | | | | | | | | | | | | |
| Hopman 54 | 2019 | | | | | | | | | | | | | | X | | | | | | |
| Doidge 29 | 2019 | | | | | | | | | | | | | | | | | | | | |
| Norris 37 | 2019 | | | | | | | | | | | | | | | | | | | | |
| Carmo 57 | 2018 | | | | | | | ? | | | NA | | | | X | | | | | | |
| Scherpenzeel 24 | 2018 | | | | | | | | | | | | | | X | | | | ? | | |
| Coyne 34 | 2018 | | | | | | | ? | | | | | | | X | | | | | | |
| Zhuo 35 | 2018 | | | | | | | | | | | | | X | | | | | | | |
| Kumar 36 | 2018 | | | | | | | ? | | | | | | ? | | | | | | | |
| Ekakoro 27 | 2018 | | | | | | | | | | | | | | X | | X | | | | |
| Hardefeldt 52 | 2018 | | | | | | | ? | | | | | | | X | | | | | | |
| Barbarossa 32 | 2017 | | | X | | | | X | | | | | | | X | | | | X | | |
| Anyanwu 25 | 2017 | | | | | | | ? | | | | | | | X | | | | | | |
| Postma 36 | 2016 | | | | | | | ? | | | | | | | X | | | | | | |
| Mc Dougall 28 | 2016 | | | ? | | | | | | | | | | | X | | X | | | | |
| Visschers 26 | 2015 | | | ? | | | | ? | | | | | | ? | X | | | | | | |
| Speksnijder 33 | 2015 | | | | | | | | | | | | | | | | | | | | |
| De Briyne 38 | 2013 | | | | | | | X | | | | | | X | X | | | | | | |
| Gibbons 30 | 2012 | | | | | | | | | | | | | | | | | | | | |
| Hughes 31 | 2011 | | | | | | | | | | | | | | X | | | | | | |

Empty cell: The study complied with this exploratory question.
Cell marked with X: The study did not comply with this exploratory question.
Cell marked with ?: We do not know whether the study complied with this exploratory question.
Cell marked with NA: This exploratory question could not be appraised in this study.

**Table S2. Quality assessment table in respect of qualitative studies: Critical Appraisal Skills Programme (CASP)**

| Author | Year | CASP criterion | | | | | | | | | |
|---|---|---|---|---|---|---|---|---|---|---|---|
| | | 1* | 2* | 3 | 4 | 5 | 6 | 7 | 8 | 9 | 10 |
| Golding 49 | 2019 | | | | | | | | X | | valuable |
| Pucken 51 | 2019 | | | | | | | | | | valuable |
| Hopman 39 | 2018 | | | | | | | | | | valuable |
| Smith 40 | 2018 | | | | X | | X | | | | valuable |
| King 41 | 2018 | | | | | | ? | | | | valuable |
| Chauhan 44 | 2018 | | | | | | | | | | valuable |
| OM 43 | 2017 | | | | | | | | | | valuable |
| Higgins 42 | 2016 | | | | | | | | | | valuable |
| Coyne 47 | 2016 | | | | | | | | | | valuable |
| Etienne 53 | 2016 | | | | | | | | | | valuable |
| Coyne 45 | 2014 | | | | | | | | | | valuable |
| Speksnijder 46 | 2014 | | | | | | | | | | valuable |
| Redding 50 | 2014 | | | | X | | ? | | | | valuable |
| Mateus 48 | 2014 | | | | ? | | ? | | | | valuable |

*Screening questions.

Empty cell: The study complied with this exploratory question.
Cell marked with X: The study did not comply with this exploratory question.
Cell marked with ?: We do not know whether the study complied with this exploratory question
10. How valuable is the research?: Valuable; Let us consider that the article provides valuable information regarding the explored research topic
